# Seed morphology uncovers 1500 years of vine agrobiodiversity before the advent of the Champagne wine

**DOI:** 10.1038/s41598-021-81787-3

**Published:** 2021-01-27

**Authors:** Vincent Bonhomme, Jean-Frédéric Terral, Véronique Zech-Matterne, Sarah Ivorra, Thierry Lacombe, Gilles Deborde, Philippe Kuchler, Bertrand Limier, Thierry Pastor, Philippe Rollet, Laurent Bouby

**Affiliations:** 1 ISEM, Univ Montpellier, CNRS, EPHE, IRD, Montpellier, France; 2 Archéozoologie et Archéobotanique, CNRS/Muséum national d’Histoire Naturelle, Paris, France; 3 Genetic Improvement and Adaptation of Mediterranean and Tropical Plants, Univ Montpellier, CIRAD, INRA, Montpellier SupAgro, Montpellier, France; 4 UMR 7041, Archéologies environnementales, Nanterre, France; 5 Archéologie Alsace, UMR 7044 Archimède, Strasbourg, France; 6 Inrap, Paris, France

**Keywords:** Plant sciences, Plant domestication

## Abstract

A crucial aspect of viticulture is finally unveiled as the historical dynamics of its agrobiodiversity are described in the Champagne region for the first time. Outline analyses were carried out to compare the morphology of archaeological grape seeds from Troyes and Reims (first c. AD to fifteenth c. AD) with that of a reference collection of modern seeds, including wild vines and traditional grape varieties, believed to be ancient and characteristic of the French vine heritage. This allows us to document the chronological dynamics of the use of the wild *Vitis* type and of the diversity of the varieties used, based on morphological disparity. After showing the existence of morphological types corresponding to geographical groups, we highlight a geochronological dynamic. Our results show that the wild type is used throughout the series, up to the Middle Ages. In addition, domestic forms, morphologically related to southern varietal groups, are very early involved in the Champagne grape agrodiversity. The groups corresponding to the typical grape varieties of today do not appear until the second millennium. These previously unsuspected dynamics are discussed in light of the social, societal and climatic changes documented for the period.

## Introduction

According to archaeological and archaeobiological data, six centuries separate the development of viticulture in Mediterranean France from the advent of Champagne vineyards, which are now famous worldwide. Champagne is sometimes regarded as “the most popular wine in the world, the best well-known, and most frequently imitated”^1^. This modern sparkling wine was invented in the seventeenth century yet the winemaking process and the definitive set of varieties used (including “Chardonnay” and several “Pinot”) were only fixed in the eighteenth century^2^.

**Figure 1:**
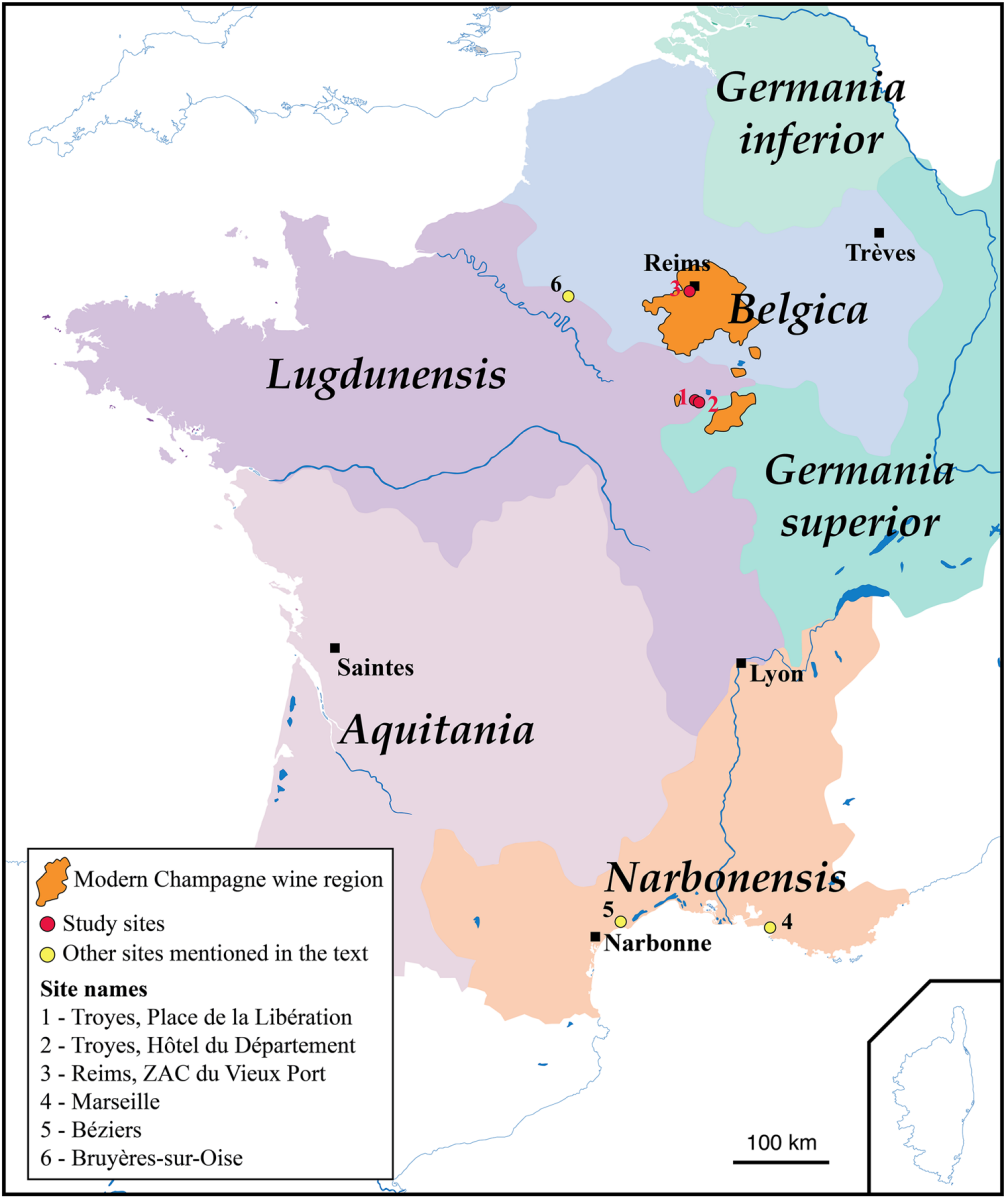
Gaul during the first century A.D.

It is a well-known fact that the Phocaeans introduced viticulture in France after the foundation of Marseille, around 600 BC (Fig. 1). During the first century BC, viticulture developed largely under Roman influence and many wine-growing establishments are documented in the Narbonnaise province, particularly around Béziers (Fig. 1). The wine produced in this region is widely exported to the Rhine valley, to Rome, and more generally throughout the Empire^3,4^.

The expansion of winegrowing from the Mediterranean to temperate Gaul (Fig. 1) is more difficult to document because amphorae, dolia, vine pressers, and masonry vats are often replaced by wooden implements and structures, such as barrels^4^. Nevertheless, recent discoveries register a prompt expansion, as early as the first century AD, and a massive development from the second century onwards in Aquitaine, Auvergne and central France. Viticulture was then practiced in Burgundy and also in the Loire and Seine valleys^5–8^. However, the establishment and development of viticulture in more northern areas, including the Champagne region, remain poorly documented. The Moselle and Rhine vineyards are well known from texts and archaeology, but they seem to develop late, from the third century onwards, and largely concentrated around Trèves, capital of Belgian Gaul and of the Empire from the end of the century onwards^4,9,10^. Between the years 20–40 AD and the third century AD, evidence of grape seeds multiplied in northern Gaul. However, these remains are almost exclusively limited to urban contexts, which suggests consumption rather than production^11^. On the other hand, very few is known about the historical agrobiodiversity of the grapevine itself despite the fact that molecular approaches can sometimes be directly applied on archaeological material^12–16^.

Morphometrics, that is the description of the shape and its covariations^17^, has led to major archaeobotanical advances, particularly for domesticated plants^18–21^. So, how much do we know about the historical agrobiodiversity of the cultivated grapevine? Recent data based on outline analyses of subfossil pips lifted the veil on the use of wild morphotypes in several key regions, as well as on the large diversity of domesticated forms exploited^22–25^.

The available studies mostly concern Mediterranean France, during the Iron Age and more particularly during the Roman period. Archaeological pips show morphological similarities with contemporary varieties including some varietal groups with very distinct geographical origins^22,25–27^. The characteristics of grapevines point to the existence of a great variety and variability between sites, which are geographically close.

The large and significant dataset of archaeobiological material assembled made it possible to challenge two conventional views: (i) the sudden replacement of wild vines by their domesticated counterpart; (ii) the low varietal diversity within vineyards. These results plead for important exchanges and contacts between regions, which is corroborated by archaeogenomics^28^. However, we still lack a finer description of cultivated diversity at both global and regional scales. As concerns the Champagne region, recent excavations in the old districts of Troyes (Place de la Libération and Hôtel du Département; dir. P. Kuchler and G. Deborde) and Reims (Boulevard Henrot/ZAC du Vieux Port; dir. P. Rollet) yielded a very well documented chronological sequence covering part of the Roman period (20–40 AD to the third century) and the Middle Ages (tenth–fifteenth centuries). Vine pips preserved in anoxic conditions were found in abundance throughout the sequence associated with other biological material. At Place de la Libération, the infilling of a well, dated from the second century, yielded an assemblage containing numerous seeds, pedicels and fragments of skins and stalks, interpreted as residues of grape pressing and winemaking^11^. However, the corresponding vineyards are difficult to locate. Furthermore, the identity of the varieties that contributed to the establishment and development of the Champagne vineyards remains unknown.

This is why, in this study, we used outline analyses on these grape seeds to explore the dynamics of the historical grapevine agrobiodiversity in Champagne. This work allowed us to estimate the proportions of wild versus domesticated types, to recognize their morphological disparities and to acknowledge their similarities to modern regional morphotypes. This will also make it possible to document temporal variations in the grape diversity exploited and discuss whether they reflect human expertise and environmental events.

## Results

### Dynamics of wild versus domesticated types

**Figure 2:**
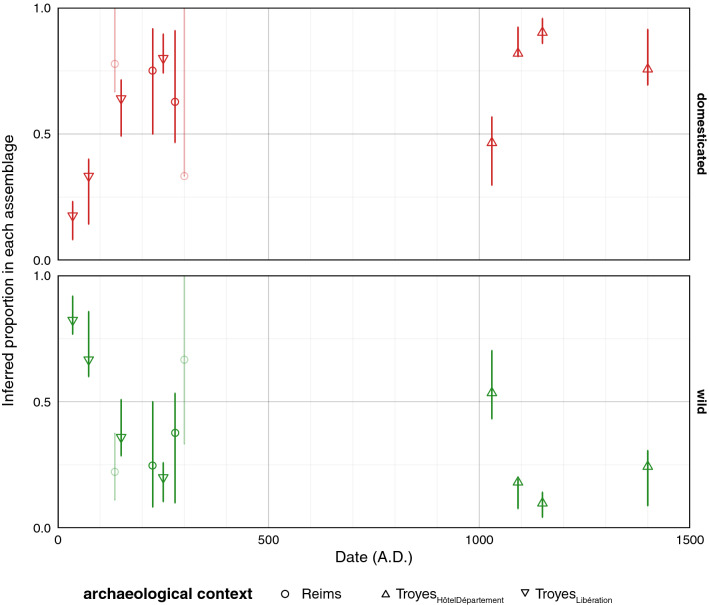
Inferred dynamics of the domesticated vs. wild type proportions. Identification were provided by LDA_status_ and proportions are calculated within each assemblage. Observed proportions are indicated with a symbol, confidence intervals are obtained through 10^3^ permutations, both are faded when sample sizes are small.

The linear discriminant analysis LDA_status_, on the specimens from the modern reference collection, led to an almost perfect discrimination between wild and domestic grapevine seeds (accuracy = 96.9%; 2297/2400 pips correctly classified). When applied to the archaeological pips, the proportion of domesticated grapevine varied from 18% (35-Troyes_Libération_) to 90% (1150-Troyes_HôtelDépartement_). The results obtained by phase are detailed in Fig. 2. During the first three centuries, this proportion of the domestic type increased from 25 to 75%. This trend is observed for all the available phases (Reims_VieuxPort_ and Troyes_Libération_). After a gap of seven centuries, 1002-Troyes_HôtelDépartement_ presented a roughly balanced proportion of wild and domesticated types. For the next three phases, the proportion of domesticated vines increased again and exceeded 75%.

### Principal component analysis of archaeological pips

**Figure 3:**
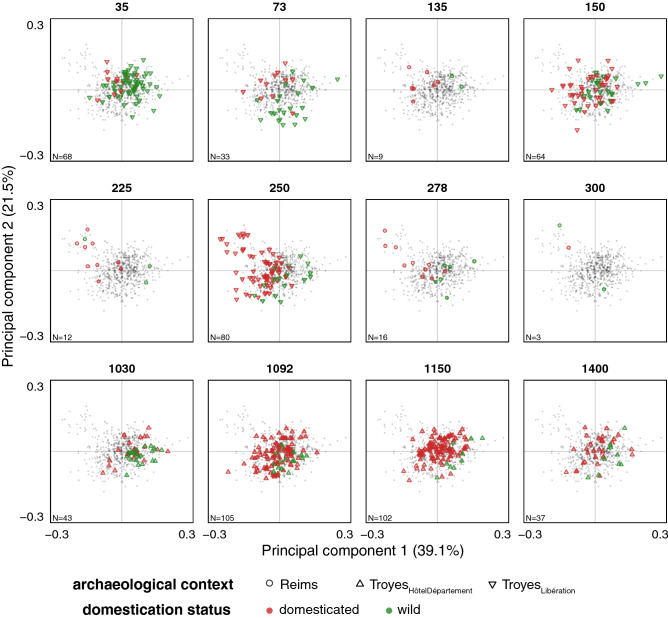
Principal component analyses on archaeological material. A single PCA is obtained (all grey spots), each facet highlights a phase, and each point corresponds to a pip. Symbols represent sites and colors report identification obtained with LDA_status_.

**Figure 4:**
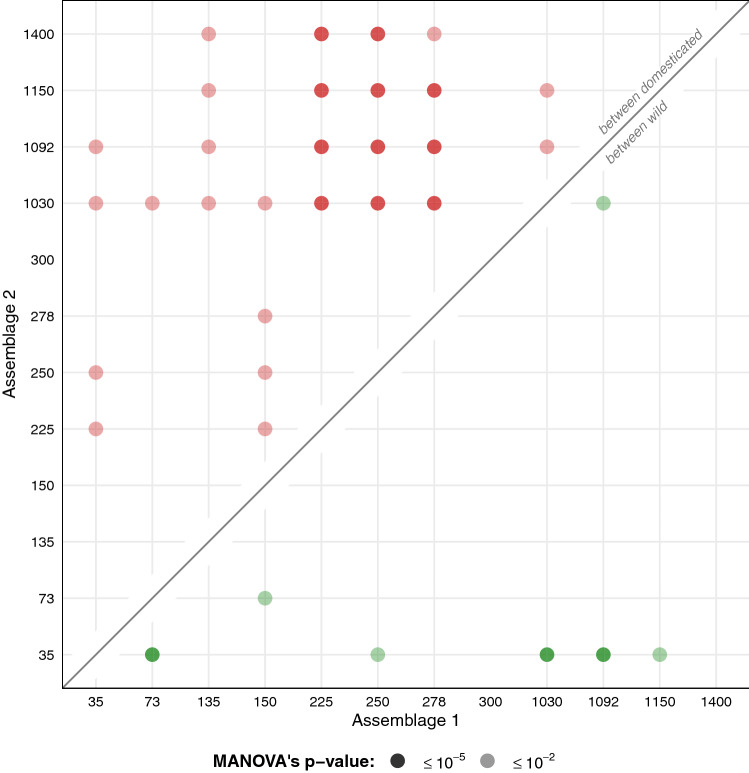
Pairwise multivariate analyses of variances. Differences were tested using the first two principal components showed in Fig. 3, between pairs of assemblages and separately for sets of pips identified as of domesticated and wild types. 300-Reims_VieuxPort_ was removed from pairwise comparisons due to very small sample size (N = 3).

The principal component analysis on the shape of archaeological seeds showed clear differences between phases (Fig. 3). The first two principal components captured 60.6% of the total shape variability (PC1 = 39.1%, PC2 = 21.5%). These first two components were used as synthetic shape variables in the pairwise MANOVAs (Fig. 4). The dimensional reduction of the PCA makes it possible to handle small sample sizes, particularly for the Reims_VieuxPort_ phases.

Among the seeds identified as domestic, the different phases of the Middle Ages (here those from the tenth century onwards) differed from those of the Roman period (second century). Concerning wild type pips, there are significant differences between the first two phases (35-Troyes_Liberation_ and 73-Troyes_Liberation_); the oldest phase of this set (35-Troyes_Liberation_) is also different from the first two phases of the second millennium (1002-Troyes_HôtelDépartement_ and 1092-Troyes_HôtelDépartement_).

### Dynamics of the morphological disparity

**Figure 5:**
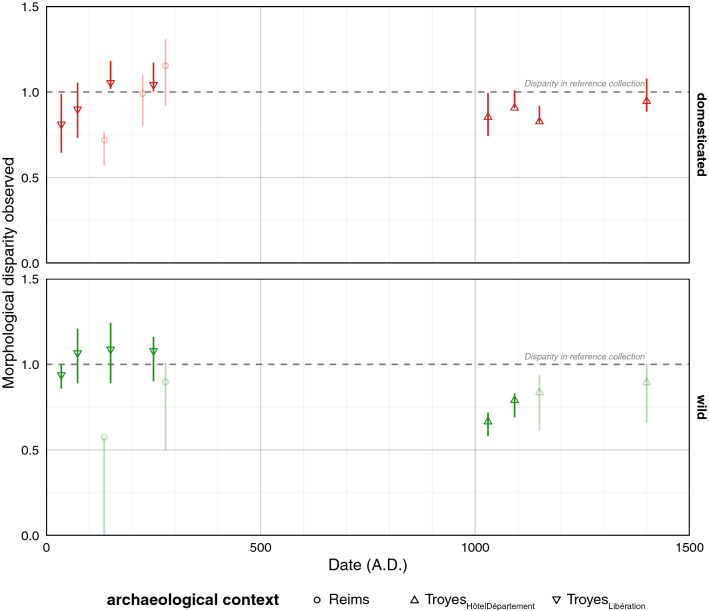
Dynamics of morphological disparity. For each compartment, disparities were standardized by the observed disparities in the reference collection. Symbols represent sites, confidence intervals were obtained through 10^3^ permutations, colors report identification obtained with LDA_status_, all are faded when sample sizes are low.

During the first three centuries AD, the morphological disparity between the domesticated groups increased and was even greater than 1 (Fig. 5). Again, this trend is consistent between the Reims and Troyes_Libération_ phases. As the disparities have been normalized by the average disparities (domesticated/wild) obtained from the entire reference collection, values greater than 1 reflect a particularly diversified sample. For the Middle Ages, the disparity is still high (but less than 1) and stays roughly constant.

Among pips of the wild type, the disparity over the first three centuries increased less markedly than for their domesticated counterparts, and is approximately equal to or greater than 1. The smallest disparities are observed for the medieval phases. Overall, these disparity values suggest that diversity increased during the first three centuries AD and remained consistently high thereafter, for both compartments.

### Inference of geographical morphotypes

The application of LDA_cépage_ on modern material led to good identification of the grape varieties (86.7%—1509/1740), and even to an excellent identification if we only retain pips with a posterior probability ≥ 0.8 (97.2%—1563/1608). Varieties ‘Pinot noir’, ‘Meunier’, ‘Pinot blanc’, ‘Gamay’, ‘Meslier Saint-François’, known to be very close parents, are the only ones showing a class precision of less than 80%. When geography is based on this identification, the precision of the geographical classes is systematically higher than 97%.

The application of LDA_region_ on the modern material led to a lower accuracy (58.8%, 1144/1740 correctly classified seeds). The filtering of pips with a posterior probability ≥ 0.8 was quite strict, since only 32% of the pips were retained. As expected, the accuracy improved (88.4%—497/562 correctly classified seeds). Class accuracies ranged from 77 to 96% (for the "East" and "South" groups, respectively).

The inferred regional origins are presented as proportions within each phase in Fig. 5. The proportion of wild type seeds is directly transferred from LDA_status_, whose domesticated/wild trends were presented before. The inferred proportions suggested that, in the early centuries, almost all seeds of domesticated type were morphologically similar to present day varieties from the "South" of France (Provence, Languedoc) and even from the "far South" (Italy, Spain and Greece). Similarly, in the phases of the third century (225-Reims_VieuxPort_, 250-Troyes_Libération_ and 278-Reims_VieuxPort_), certain seeds are attributed to groups from the "West" (Bordeaux, Loire), the "South West" and the "East" (mainly Germany and Alsace-Lorraine). Finally, the seeds identified as belonging to the regional groups of Champagne and Burgundy did not appear until the eleventh century, again in association with representatives of the "East" group.

## Discussion

The archaeological grape pips analyzed here form a quite exceptional dataset in that they cover fifteen centuries of viticulture (first to fifteenth c. AD) in the Champagne region. Albeit with an important hiatus. What can these pips tell us about the grapes used to make wine? Was there a great diversity of varieties? If so, where did they come from? Is there a temporal dynamic? If so, does it reflect anthropogenic and environmental events?

### Early adoption of cépages, late persistence of morphologically wild grapes

The first unexpected result is the omnipresence of morphologically wild grapes up to the fifteenth century (Fig. 2). Assuming that the relative proportions of wild and domesticated types reflect their relative use, we can consider that the exploitation of the wild type decreased sharply during the first centuries of our era. Similarly, the domestic grapevine is used from the first century AD onwards, but is not dominant until the second century.

We know now that the wild morphotype is ubiquitous in the Roman sites of the Narbonnaise, in such proportions (more than one third in most cases) that it seems unlikely that it represents only gathered berries from wild vines. The presence of the wild grapevine is attested nowadays^29^ and in the Roman times^30^ in the area. It seems possible that this wild morphotype might correspond to a particular type of cultivated grapevine^25,26,31,32^. We could be dealing here with wild vines directly introduced into cultivation for some desirable properties or domesticated types whose morphology is still only slightly influenced by selection processes. It seems that the Romans cultivated a continuum of diversity between wild types and vines corresponding to different domestic types still exploited today. In the South, the use of the wild morphotype decreases during the second part of the Roman Empire^31^ while in Champagne, this decrease is recorded from the first to the second c. AD.

For the Medieval period, the first sample (eleventh century) is again mostly composed of the wild type which decreased again during in the fourteenth to fifteenth centuries. For the time being, it is difficult to explain this result. The seven-century hiatus (from the third to the tenth century) does not help our attempt to shed light on this question. However, both written documents and archaeobotanical data agree that viticulture was maintained in north-eastern France, from the Roman period to around 1000 AD^9^. Two main hypotheses should therefore be considered. First, it is possible to imagine that during times of climatic or economic crisis, people might feel obliged to re-exploit the wild morphotype, during times of climatic or economic crisis before the emergence of a new set of better adapted varieties. Another hypothesis considers the importance of pests or parasites, which could have been introduced with foreign varieties, and thus be partly responsible for the decline and re-compositions of the grapevine diversity^33^. For the moment, no hypothesis can be privileged before more data are available especially for the Late Roman and Early Medieval period.

### Morphological disparity: a parsimonious approach and congruent patterns

The morphological disparity approach does not formulate a priori neither on grape varieties, nor on their supposed geographical origins. Although not perfect, only this approach allows us to directly "measure" past agrobiodiversity. Disparity results show trends that resonate with the wild versus domesticated dynamics discussed above (Fig. 4). For the Roman period, this diversity increases for domesticated grape varieties and remains constant for the wild morphotype; for the Middle Ages, neither wild-type vines nor grape varieties "return" to previous levels.

Finally, another parsimonious approach is that of differences between morphological assemblages, tested here after a dimensional reduction (Fig. 3), given the small size of the sample. The results are also consistent and show that the phases of the Middle Ages differ from those of the Roman period, especially concerning seeds of the domesticated type.

### Geographical morphotype fluctuations in pip assemblages

The history, the geography and the genealogy of grape varieties are inextricably intertwined. If molecular approaches can cut the Gordian knot of genealogy^34,35^, history and geography must rely on other sources. The two methods used here to test the existence of geographic morphological types have acceptable accuracy before filtering (LDA_cépage_ = 87% and LDA_region_ = 59%) and excellent accuracy after filtering (97% and 88%). These results indicate that "geomorphotypes" do exist, i.e. the geographical origin of a grape variety can be inferred from the shape of its seed.

**Figure 6:**
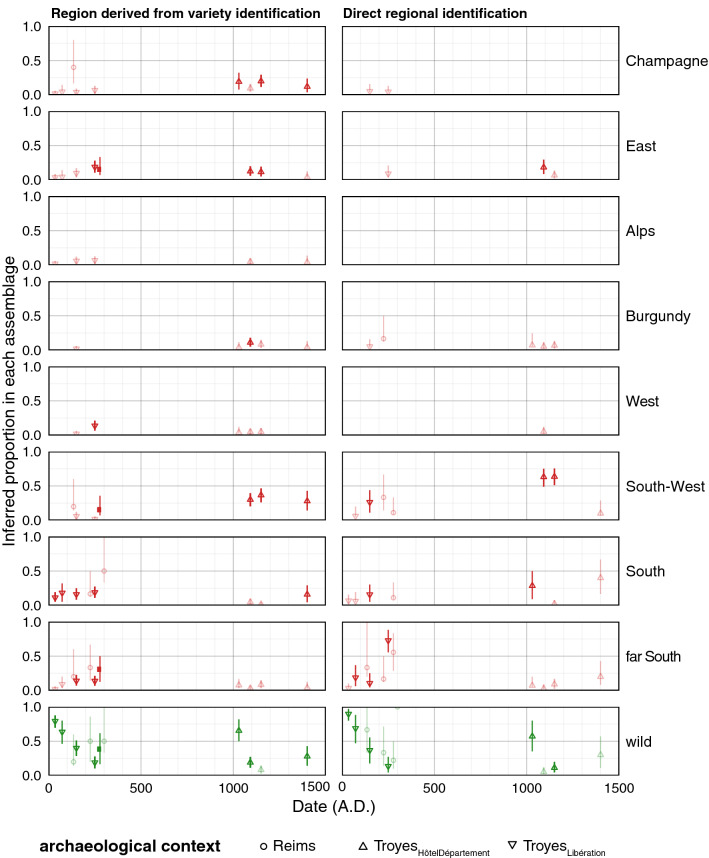
Dynamics of regional origins in each phase. The two columns report regional origins obtained through LDA_cépage_ and LDA_region_; rows report proportion of geographical groups calculated within each phase. Symbols represent sites, confidence intervals were obtained through 10^3^ permutations, colors report identification obtained with LDA_status_, all are faded when sample sizes are low.

Applied to archaeological material, these two methods provide pips with a geo-historical affiliation (Fig. 6). These two approaches are largely congruent and of unparalleled resolution. They show that the Champagne pips from the first two centuries AD have strong morphological affinities with the current types from the south (South-West, South, far South), to the exclusion of all others. The third century is characterized by the appearance of grape varieties with seeds of different shapes, typical today of the West (Bordeaux and Loire) and the East (Germany, Alsace-Lorraine). In the eleventh and twelfth centuries, this pattern changes as the presence of Burgundian and Champenois types is recognized for the first time, but the South Western and Extreme-Southern types are still recorded in unequalled proportions on the series.

These regional identifications are rather effective from a statistical point of view. Of course, a LDA returns an identification based on the level with the highest posterior probability but this indicates morphological proximity, not varietal identification. For instance, two varieties may be morphologically homologous (for example, if they are direct or close relatives) or analogous (if the similarities are fortuitous and not inherited from a common parent). That being said, a non-random regional grouping exists within our reference collection built up to represent old grape varieties of different regions.

We believe that these subtle differences in shape, partly associated with regional differences, are non-adaptive for the grapevine; they might be co-products (e.g. through genetic hitchhiking) of human selection for other traits, more directly related to cultivation and winemaking. These differences nevertheless document a geographic-historical grey area in the history of French viticulture.

### Which social and climatic factors can explain the observed fluctuations?

Our results concern two major periods, Roman and Medieval, for historical Champagne viticulture, separated by a gap of seven centuries due to the lack of documentation.

The Roman period (first to second centuries AD) is marked by the declining use of the wild morphotype and the early adoption of grape varieties. However these first varieties are of meridional affinities which contrasts with the historical scheme proposed by Dion in 1959; according to this author the extension and specialization of vineyards in temperate Gaul was only made possible by the creation of varieties resistant to either oceanic moisture or continental climates.

The medieval period is first marked by the unexpected late revival of the use of the wild morphotype which even predominates during the eleventh century. In addition, varieties typical of today's Champagne region also appear for the first time. However, southern types are still clearly predominant in the eleventh and twelfth centuries. How can this predominance be explained?

It is now clear that climate is one of the main drivers of the evolution of societies and the development of agriculture^36,37^ and viticulture in Champagne does not escape this trend. Medieval Europe experienced an unusually warm period, the "medieval climatic optimum", from around 950 to 1350^38^. The estimated amplitude of the change, an increase of a few tenths of a degree in the average temperature, may seem minimal, but it is significant enough for the grapevine and winemaking. For instance, current changes are affecting yield for both red and white varieties but also grape composition with changes in sugar and acidity concentrations, as well as polyphenols and aroma compounds^39,40^. This period corresponds in fact to the maximum northern extension of viticulture, to southern England and the shores of the Baltic^41^. This medieval climatic optimum is also recognized in the dates of the harvest, which provide the longest continuous record of the phenology of the vine, and more broadly of past climate^42^. This climatic warming may explain the presence of southern types at high latitudes. This favourable climatic period is also, a period of economic change and demographic growth^43^, described by Duby as the "agricultural revolution of the Middle Ages^44^". The evolution of agricultural techniques and practices made it possible to increase land clearing for agriculture, which in turn stimulated and promoted economic and demographic growth.

This warm period was followed by the "Little Ice Age"^45^, which was probably a key factor in the region's viticulture shift: non-native grape varieties, planted beforehand to establish the Champagne vineyard, decline and are replaced by northern varieties, either newly obtained or already present in the vineyards; these varieties would be better adapted to the environmental conditions of the thirteenth and fourteenth centuries AD. These climatic events probably determined, at least in part, the modern Champagne vine stock.

This new data lifts the veil on the agrobiodiversity of Champagne viticulture from the first to the fifteenth century AD. Grape pips with wild-type morphology are omnipresent, even in the latter moments; the diversity of the domestic vine is extensive, even at the early stage of local viticulture, and presents morphological similarities with current varietal groups from different geographical origins. Our results suggest that the history of viticulture in Champagne may be closely linked to historical events and past global climatic changes. Although limited to the Champagne vineyard, this study underlines the interest of applying contour analyses to archaeological pips and paves the way to obtaining a more global corpus that will allow a better understanding of the history of viticulture as a whole.

## Methods

### Modern reference collection

Our approach aims to shed light on archaeological data using a modern reference collection. This study includes 79 *accessions* of *Vitis vinifera* L. divided into 57 varieties (or *cultivars*, or *cépages*) of domesticated grapevine (*Vitis vinifera* L. subsp. *vinifera*) and 22 individuals of wild grapevine (*Vitis vinifera* subsp. *sylvestris* (C.C.Gmel.) Hegi) collected in 12 locations that cover the current distribution of this endangered (sub)species (Table 1). Grapes were collected at maturity, both in their natural habitat for the wild individuals, and in the French central ampelographic collection for the domesticated varieties (INRAE, Centre de ressources biologiques de la vigne de Vassal-Montpellier; https://www6.montpellier.inrae.fr/vassal). For each accession, 30 normally developed berries were collected randomly from fully mature bunches. Therefore, the modern reference collection used includes 2370 pips.

**Table 1:** List of wild individuals and domesticated varieties in the modern reference collection.

Cultivar name	Presumed origin	Geographical origins	Historical typology
**Cépages (Vitis vinifera L. subsp. vinifera)**			
Aligoté	France, Bourgogne	Burgundy	NA
Arbane (syn. Fromenteau)	France, Loire	Champagne	Ancient
Arvine	Suisse, Valais	Alps	Primitive
Bachet	France, Aube	Champagne	Ancient
Barbera	Italie, Piémont	Far South	NA
Bourboulenc	France, Provence	South	Ancient
Bouteillan (syn. Colombaud)	France, Provence	South	NA
Cabernet franc	France, Aquitaine	West	Ancient
Cabernet-Sauvignon	France, Aquitaine	West	Modern
Calitor	France, Provence	South	NA
Carignan	Espagne, Aragon	Far South	Ancient
Chardonnay	France, Bourgogne	Burgundy	Modern
Chasselas	Bourgogne/Suisse	Alps	Ancient
Chenin	France, Pays-de-Loire	West	Ancient
Cinsaut	France, Provence	South	Ancient
Clairette	France, Languedoc	South	Ancient
Cot (syn. Malbec)	France, Sud-Ouest	South West	NA
Dono d'Enrico (syn. Doux d'Henry)	Italie, Piémont	Far South	NA
Duras	France, Sud-ouest	South West	Ancient
Dureza	France, Rhône-Alpes	Alps	NA
Frankenthal (syn. Schiava grossa)	Allemagne/Italie	East	Ancient
Gamay	France, Bourgogne	Burgundy	Modern
Gouais (syn. Heunisch Weiss)	NA	East	Ancient
Grenache (syn. Garnacha)	Espagne	Far South	Ancient
Grüner Verteliner	Autriche	East	NA
Humagne	Suisse, Valais	Alps	Ancient
Len de L’el	France, Midi-Pyrénées	South West	NA
Mauzac	France, Midi-Pyrénées	South West	Ancient
Melon	France, Bourgogne	Burgundy	NA
Merlot	France, Aquitaine	West	Modern
Meslier Saint François	France, Gâtinais	Champagne	Modern
Meunier (syn. Pinot meunier)	France, Bourgogne	Burgundy	Ancient
Mondeuse blanche	France, Rhône-Alpes	Alps	Ancient
Mourvèdre (syn. Monastrell)	Espagne	Far South	Ancient
Muscat à petits grains	Grèce	Far South	Ancient
Negrette	France, Sud-Ouest	South West	NA
Petit Meslier	France, Est	Champagne	Ancient
Petit Verdot	France, Aquitaine	South West	Primitive
Peurion	France, Bourgogne	Burgundy	Ancient
Pinot noir	France, Bourgogne	Burgundy	Ancient
Pinot blanc	France, Bourgogne	Burgundy	Ancient
Piquepoul blanc	France, Languedoc	South	Ancient
Précoce Bousquet	France, Sud-Ouest	South West	NA
Primitivo (syn. Zinfandel)	Croatie	East	Ancient
Riesling	France, Est	East	Ancient
Rivaïrenc (Aspiran blanc)	France, Languedoc	South	NA
Roussaïtis	Greece	Far South	NA
Samoriau	France, Bourgogne	Burgundy	NA
Sauvignon	France, Centre/Sud-Ouest	West	Ancient
Savagnin blanc (syn. Traminer)	Italie, Tyrol	East	Primitive
Syrah	France, Rhône-Alpes	Alps	Modern
Tibouren	France, Provence	South	NA
Tressot	France, Sud-Ouest	South West	Ancient
Troyen	France, Nord-Est	Champagne	Modern
Ugni blanc (syn. Trebbiano toscano)	Italie, Toscane	Far South	Ancient
Vermentino	Italie	Far South	NA
Viognier	France, Rhône-Alpes	Alps	NA

Locations and identifiers	Geographical origin		
**Wild individuals (Vitis vinifera subsp. sylvestris (C.C.Gmel.) Hegi)**			
El Centenillo 2, 5, 9	Espagne, Andalousie		
Grésigne E, F	France, Tarn		
Ile de Ketsch 3, 8, 11	Allemagne, Baden-Würtemberg		
La Vall 3	France, Pyrénées-Orientales		
Lago di Martignano 1	Italie, Province de Rome		
Olave 2, 3	Spain, Communauté valencienne		
Pic Saint Loup 12, 13	France, Hérault		
Salvan 1, 2	Suisse, Valais		
Sarantaporos 2	Grèce, Thessalie		
Saint Croix en plaine 1	France, Haut-Rhin		
Valbonne 1	France, Pyrénées-Orientales		
Venetikos 1, 2, 11	Grèce, Macédoine occidentale		

Geographical origins and historical typology are derived from Thierry Lacombe (2012). All varieties are wine varieties with the exception of “Chasselas” and “Clairette”, which have mixed table/wine uses. NA indicates non available information. Wild individuals are provided with their location and alphanumerical identifiers.

The varieties included are characteristic of the main French wine-growing regions as well as of geographically close foreign vineyards; most of them are considered traditional or old varieties, mentioned in documents written before the phylloxera crisis. All varieties are dedicated to winemaking yet the varieties “Chasselas” and “Clairette” are also appreciated as table grapes.

### Archaeological corpus

Preventive archaeological excavations carried out at Troyes (Place de la Libération and Hôtel du Département) and Reims (ZAC du Vieux Port) enabled the investigation of (sub)urban contexts, particularly wells, in which anoxic conditions had contributed to the exceptional conservation of biological material, including grape seeds, sometimes associated with pedicels, skin and leaf fragments. In each site, four chronological phases were studied, each phase corresponding to a unique archaeological context. In general, the archaeobotanical assemblages were made up of a mixture of different wastes, so they probably include grape seeds of multiple origins, which may correspond to fruits harvested over several years. Nevertheless, all these assemblages come from closed archaeological structures such as wells or basins. Their filling is thus well protected from post-deposit perturbations. The study of the archaeological material (ceramics, coins, etc.) confirms that these layers are undisturbed, that they correspond in each case to a single phase of accumulation that can be well and safely dated by the archaeological material. In two cases where the archaeological material was not characteristic enough to provide a reliable dating, two grape seeds were directly dated by radiocarbon.

The site of the Place de la Libération in Troyes is located in the center of the Roman city. The assemblages related to the first two phases of the site (20–50 AD, 60–85 AD) come from a basin and a well, respectively. At that time, this part of the city was a residential area. The sample from the phase 100–200 AD comes from a well associated with warehouses related to the storage and trade of wine. The well contained grape pressing residues composed of grape pips, pedicels and skins. The last assemblage (200–300 AD) came from an urban ditch filled with rubbish deposits.

The second site of the city of Troyes, Hôtel du Département, corresponds to a tanners' district between the twelfth and nineteenth centuries AD. The archaeobotanical assemblages originate from two pits, a cesspit and a barrel, all used as secondary rubbish dumps. All these samples date back to the Medieval period.

The last site, ZAC du Vieux Port, is located in the city of Reims, which was the capital of Belgian Gaul under the rule of Emperor Augustus. The area excavated provided evidence of living and artisanal quarters as well as piers on the banks of the river Vesle, all related to the Roman period. Archaeobotanical sampling was carried out in rubbish deposits from gutters and in the river bed. Samples cover the period second to fourth centuries AD. A total of 572 waterlogged, unbroken and well-preserved seeds were recovered, analyzed and compared with the modern reference collection (Table 2, Supplementary Figure 1). The archaeological layers that yielded the vine remains are mainly dated by the archaeological artefacts (ceramics, coins) but, when needed, radiocarbon dating was also carried out directly on seeds (Table 2). For the sake of simplicity, the mean value of all dating intervals is used in subsequent analyses and graphs.

**Table 2:** Archaeological corpus. Sites and dates for material included in this study.

Archaeological site	Dating	Dating method	Phase and coding	Number of pips
Troyes, Place de la Libération	20–50 AD	Dendrochronology, Archaeological artifacts	35-Troyes_Libération_	68
Troyes, Place de la Libération	60–85 AD	Dendrochronology, Archaeological artifacts	73-Troyes_Libération_	33
Reims, ZAC du vieux Port	110–160 AD	Archaeological artifacts	135-Reims_VieuxPort_	9
Troyes, Place de la Libération	100–200 AD	Dendrochronology, Archaeological artifacts	150-Troyes_Libération_	64
Reims, ZAC du vieux Port	210–240 AD	Archaeological artifacts	225-Reims_VieuxPort_	12
Troyes, Place de la Libération	200–300 AD	Dendrochronology, Archaeological artifacts	250-Troyes_Libération_	80
Reims, ZAC du vieux Port	255–280 AD	Archaeological artifacts	278-Reims_VieuxPort_	16
Reims, ZAC du vieux Port	280–320 AD	Archaeological artifacts	300-Reims_VieuxPort_	3
Troyes, Hôtel du Département	Poz-68076 : 945 ± 30 BP (1025–1157 AD)	Radiocarbon	1002-Troyes_HôtelDépartement_	43
Troyes, Hôtel du Département	Poz-68075: 1030 ± 50 BP (892–1152 AD)	Radiocarbon	1092-Troyes_HôtelDépartement_	105
Troyes, Hôtel du Département	1100–1200 AD	Archaeological artifacts	1150-Troyes_HôtelDépartement_	102
Troyes, Hôtel du Département	1300–1500 AD	Archaeological artifacts	1400-Troyes_HôtelDépartement_	37

### Statistical analyses

All analyses were performed in the R 4.0.0^46^, with the packages: Momocs 1.3.1 for morphometrics^47,48^; MASS 7.3.51.6 for discriminant analyses and cross-validation^49^; and the tidyverse ecosystem 1.2.1 for general data manipulation and visualization^50^.

### Outline analyses using elliptical Fourier transforms

All the seeds, archaeological and modern, were photographed according to two orthogonal views (dorsal and lateral), by the same operator (TP).

Outline coordinates (x; y) were extracted from these images, then centered, scaled using the centroid size, aligned and normalized for the position of their first points. For each view, elliptical Fourier transforms were used to convert the contour geometry into "Fourier coefficients". The number of harmonics retained assembled 95% of the total harmonic power which corresponded to 7 for the dorsal view and 8 for the lateral view. With four coefficients per harmonic, 60 Fourier coefficients were used as quantitative variables describing the shape (which is the form minus the size) for all subsequent analyses. For an extended description of elliptic Fourier transforms please see^47,51,52^. The full datasets used in this study are published in figshare (10.6084/m9.figshare.12987683).

### Inference of the domestication status

A first linear discriminant analysis (further abbreviated "LDA"), was applied to the 2370 seeds of the modern collection, to identify the status (wild/domestic) of the 572 archaeological seeds (LDA_status_ after). The accuracy of this LDA_status_ was calculated on the modern material, then used to predict the status of archaeological pips which were assigned to either the wild or the domestic compartment. The proportion of each type within each assemblage is then calculated. Within each assemblage, pips of each type (wild/domestic) were treated separately, in all subsequent analyses^23,53^.

### Testing differences in morphological assemblages between phases

We then applied a principal component analysis (further abbreviated "PCA") to the archaeological pips to explore how the assemblages varied between phases in terms of morphology. This PCA allowed us to reduce the size of the dataset, prior to multivariate analyses of variance ("MANOVA" below) testing the differences between morphological assemblages between phase pairs, within the wild and domestic morphotypes. The 300-Reims_VieuxPort_ was removed from pairwise comparisons due to very small sample size (N = 3).

### Inference of morphological diversity

Morphological disparity is used as an approximation of total diversity^54–57^. The morphological disparity index was here calculated on Fourier coefficients for each set of pips of a given status, in a given phase (such as displayed in Fig. 3). We used the mean of absolute distances of each pip to the centroid of each set. This measures how morphologically variable are each set of pips^58^. This approach makes it possible, although indirectly, to infer agrobiodiversity, without external assumptions.

The morphological disparities obtained are standardized, for main morphotypes, based on the disparities calculated on the whole of the modern reference collection (wild or domesticated specimen). This index is strictly positive; if higher than 1, this indicates that the assemblage is morphologically more disparate than the corresponding assemblage of the modern collection.

### Inference of regional morphological types

Two final discriminant analyses (LDA_cépage_ and LDA_region_) were used to test the existence of morphological groupings providing geographic information, i.e. the existence of "geomorphotypes". These two analyses only concerned seeds identified as domesticated. LDA_cépage_ first identifies the most similar modern variety, then infers the corresponding region, while LDA_region_ directly identifies the regional grouping (Table 1). Please note that LDA_cépage_ was only conducted as an intermediate step towards the identification of geomorphotype; morphological resemblance alone can not grant the homology, that is evidence of correspondence to any modern variety. These two approaches are independent, thus making it possible to discuss their congruence.

For both status and geomorphotype inference, identifications associated with a posterior probability greater than or equal to 0.8 are retained. Then, if within a phase, the size of the remaining sample is at least equal to 10, the proportions of each of these geomorphotypes are calculated and discussed, otherwise they are only presented and faded on the corresponding figures.

### Robustness assessment using permutational approaches

For all LDAs, we estimated the robustness of our inferences with 10^3^ resamples with replacement, within each phase. Within each combination for which a statistic is obtained, we therefore present its distribution obtained over 10^3^ resampling rather than the actual statistic observed.

## Supplementary Information

Supplementary Figure 1.

## Data Availability

Datasets used in this study are available as .rda and .yaml files there: https://figshare.com/s/02125bd2ded38ca56773 (will be made public upon acceptance).
