# Supplementary material for: Seed morphology uncovers 1500 years of vine agrobiodiversity before the advent of the Champagne wine
Source: Sci Rep. 2021 Jan 27;11:2305. doi: 10.1038/s41598-021-81787-3 (PMC7840976; doi:10.1038/s41598-021-81787-3)
Supplement: Supplementary file 1 — Supplementary Figure 1. [file 41598_2021_81787_MOESM1_ESM.docx]

**Seed morphology uncovers 1,500 years of vine agrobiodiversity**

**before the advent of the Champagne wine**

Vincent Bonhomme^1, &, @^ , Jean-Frédéric Terral ^1, &^, Véronique Zech-Matterne^2^,

Sarah Ivorra^1^, Thierry Lacombe^3^, Gilles Deborde^4^, Philippe Kuchler^5^, Bertrand Limier^1^, Thierry Pastor^1^, Philippe Rollet^6^ et Laurent Bouby^1^

^1^ ISEM, Univ Montpellier, CNRS, EPHE, IRD, Montpellier, France.

^2^ Archéozoologie et Archéobotanique, CNRS/Muséum national d’Histoire naturelle, Paris, France

^3^ Genetic Improvement and Adaptation of Mediterranean and Tropical Plants, Univ Montpellier, CIRAD, INRA, Montpellier SupAgro, Montpellier, France

^4^ UMR 7041, Archéologies environnementales, Nanterre

^5^ Archéologie Alsace, UMR 7044 Archimède

^6^ Inrap

^&^ these authors contributed equally to this work

^@^corresponding author: bonhomme.vincent@gmail.com +33 (0)4 67 14 41 60


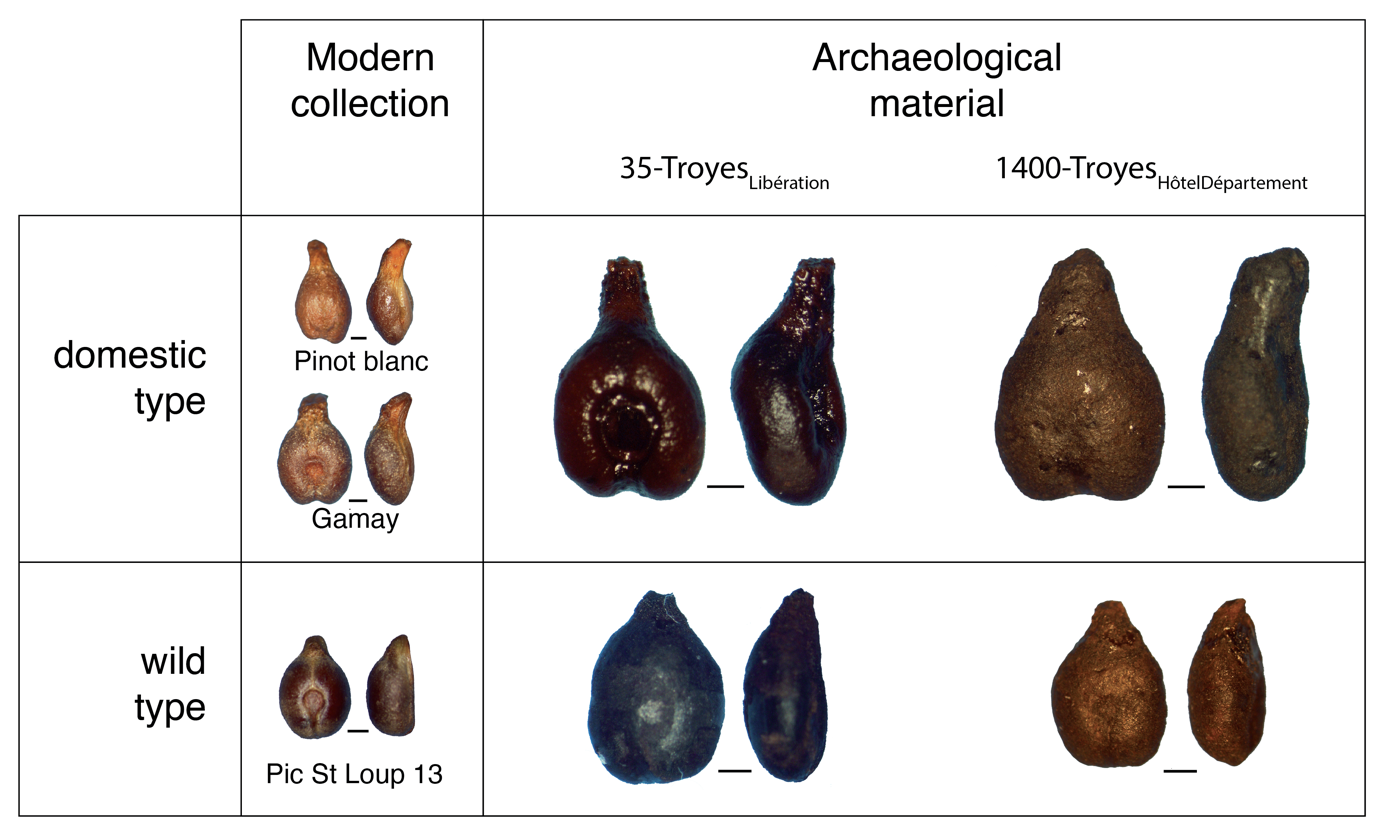


Supplementary figure 1: Some pips from the modern collection and archaeological material used in this study. Black scale bars represent 1mm.
